# Dentition and Its Derangements

**Published:** 1863-03

**Authors:** A. Jacobi


### Selections.

DENTITION AND ITS DERANGEMENTS.—*By A. Jacobi, M. D. LECTURE XII.*—PART II.—*Why Essential, or Dental, Paralysis is considered a Spinal Disease—Why Dental Paralysis is not always a Spinal Disease.*

The reasons why Dr. Heine feels justified in considering infantile paralysis as spinal, are derived from the real or supposed peculiarities of such cases in their last stage. They are, in his own words, the following:

1. The cerebral functions of the patients are entirely intact. No case of his exhibited any disturbance in either mental or sensory actions. Wherever there were any cerebral symptoms in the beginning, they disappeared rapidly.

2. Paralysis followed immediately the symptoms of a general and central disease, as fever, convulsions, congestion. Paralysis from peripheric causes shows frequently the reverse, as there is an interval between the first attack and the last stage, viz. that of paralysis.

In the commencement of essential paralysis, contraction is never observed; the limbs are perfectly paralytic, and paralysis takes place in all the affected parts at the very same time; it has, in the first period, a tendency to gradual decrease, but never to progress. Both arms are not affected at the same time, nor are arm and leg of the same side; but always either both legs, or one arm, or one leg. Affection of the trunk is not unfrequent; it produces paralytic scoliosis: in such cases the motory nerves of the lumbar and sacral plexuses of the corresponding side, together with other spinal nerves, are suffering. Where but a single arm is paralysed (a rare case indeed) the affection has its seat in the brachial plexus of the corresponding side; in most of these cases all the muscles are affected. Cases of transverse paralysis are very rare occurrences. The sensory organs are hardly affected, except in the very commencement, and then, too, but slightly. There is no pain in the secondary period.

3. Frequently it is met with in, and limited to, the two lower extremities. Hemiplegia, paralysis of but one side or extremity, is frequently the last remainder of paraplegia.

4. It is very intense. In many cases the muscles of the trunk are also paralysed; this affection may generally diminish, but the spinal character of the paralysis will be shown by the characteristic scoliotic curvature, and sometimes by the enormous deformity of the whole trunk, which differ considerably from other cases of scoliosis, and exhibit a decidedly paralytic character.

5. In this paralysis, the atrophy of the affected limb is considerable, its temperature decreases fast and very much indeed. These symptoms are not so decided in motory paralysis depending on diseases of the brain; and moreover, Prof. Budge has proved by direct physiological experiments made on the spine of rabbits, which he cut for this purpose, the influence of the spine on the temperature of the body. The decrease of temperature is more pronounced in the periphery than near the centre; it has been observed to be as low as sixty-three and a half degrees. Motory power, nervous influence, and circulation are considerably diminished, and therefore the lower temperature is readily explained. Arteries and veins have been found smaller than normal; to such a degree, even, this diminution of size and lumen may proceed, that Hutin has a case in which a number of smaller blood vessels had entirely disappeared. The diagnosis from wasting palsy (*atrophie musculaire progressive*, Cruveilhier) is established by the fact, that in wasting palsy the atrophy is the primary suffering of which paralysis is the natural consequence; whereas, in essential paralysis the atrophy is secondary, and brought on by the diminution of both nervous influence and circulation of the blood.

6. In paralysis of an arm, which sometimes would set in with the same symptoms as those described above, the post-mortem examination has proved a material alteration of that portion of the spine from which the brachial plexus takes its origin.

7. There is a total want of galvanic reaction and electrical contractility in the paralysed muscles, in infantile paralysis. The experiments of Duchenne and others yield a negative result in spinal paralysis, while in cerebral paralysis the sensibility is always intact, and sometimes even increased to painfulness. Nor does peripheric paralysis participate in this peculiarity of the spinal form. These differences have often been used for obtaining a correct diagnosis of the local cause of the disease.

8. All the authors agree in the assumption of the spine being affected in most paraplegias, in which standing erect, and walking, is rendered impossible. While now, paraplegia must be supposed to depend on a general and thorough bilateral affection of the spine or its membranes; our cases of essential paralysis give rise to the assumption of a more local, or universal alteration.

9. Essential paralysis is incurable; peripheric paralysis is not so.

10. Finally, the aspect of such patients, and the drawings taken from them, give the impression of a deep seated disease of the nervous centres, perhaps even the spine.

Such are, in the opinion of our author, the leading pathognomonic qualities of dental, essential, or "spinal infantile" paralysis. I am, however, so far from assuming them to be indisputably correct, that I think it necessary to answer several questions, of which one is this: whether essential paralysis is really a disease of the dental period, and another, whether indeed it is always the result of a disease of the spine.

We have been taught, and experience proves, that the first onset of the disease resulting in essential paralysis, may show great differences both in its symptoms, and in its course. Sometimes the final result is produced unexpectedly fast; sometimes, however, the predisposing cause requires some time to bring on the necessary alterations. For certainly, we require of necessity, permanent local alterations to explain permanent paralysis. The principal symptoms of the first attack which I have mentioned before, belong either to the brain, or the spine, or the nerves, and from this fact, contrary to the assertions of the author, we should feel obliged to conclude that the subsequent paralysis would be either of a cerebral, or spinal, or peripheric character. For the simplest facts of the pathology of the nervous system show, that like convulsions, paralysis may have its primary seat either in one of the nervous centres, or in the course, or in the periphery of a nerve. If paralysis with the same symptoms as the dental or essential form, participates in the general characters of paralysis, if further the symptoms of the first attack are either cerebral, or spinal, or peripheric, are we justified in assuming the spine to be the only seat and cause of the consecutive paralysis? At all events, the dictatorial words of the author, that "cerebral symptoms may be present in the beginning, but are not connected with the paralysis, and *must not* be brought into any etiological relation to essential paralysis," appear too little based on the generally known facts of physiology. It is not true, indeed, that in cases where paralysis is ushered in with symptoms of cerebral irritation, while these symptoms disappear afterwards and leave the mental faculties intact for the future, the brain is not affected. Those cases of paralysis in which this very paralysis was the prominent, or rather only symptom during life, and the post-mortem examination revealed some of the traces of recent or old apoplexy in the cerebral substance, as for instance, encysted remnants on the dura mater, in both adults and children, ought to be satisfactory proofs to the contrary. At all events, I am a little slow in believing in the actual correctness of the remark of our author, that the premonitory cerebral symptoms are usually slight, but that in very violent attacks life may be endangered, although fatal termination without complications has not been observed *by him.* I have no doubt that this last addition is correct. For institutions like his, receive their patients after the disease has been allowed to remain a shorter or longer period, when the narratives of the relations are the only guides of the specialist; and indeed, such patients as are brought into an institution for the performance of tenotomy, and the application of extending apparatus, etc., do not belong to the number of those who died in the first attack. If the doctor meant to say that, it was a superfluous undertaking.

You have heard that but few post-mortem examinations of old cases of essential paralysis are on record. In some, local alterations inside the vertebral column have been found, in others not. Dr. von Heine takes those in which the results of extravasations or exudations were visible, as spinal. Good. Those in which nothing has been found, however, he also considers as spinal, because either microscopical examinations (which have not been made or have given no result) *might* have afforded a proof, or because there are cases in which paralysis is not explained by any pathological changes. The latter he takes as spinal; thus for instance a case of Longet's in which the examination resulted in finding no spinal disease, but atrophy of the roots of some nerves. Nor is he less bashful in explaining away pathological reults. Thus a case of Behrend's in which both brain and spine are described as diseased, is also crowded into the spinal flock of paralyses.

You see after all, that not in every case of dental or essential paralysis a diagnosis can be made, inasmuch as the differential diagnosis of the first insults could not be, or was only made during, or from the former periods of the disease. But always be sure to bring into account not only the spine, but be aware that diseases of all the nervous centres and motory nerves may be the causes of paralysis; on the only condition that they give rise to alterations essentially injuring the action of the nerves. It is true, further, that many diseases are more frequent in a certain age; and that the symptoms, in spite of the same locality and character, may differ according to age and individuality; but this certainly ought not to influence and prejudice us so far as to multiply our classifications and varieties. For prejudice when once allowed to prevail in pathology, will lead to further extravagances. Thus Dr. von Heine means to simply exclude the cases commencing with cerebral symptoms, terminating in paralysis, and finally recovery, from the number of the dental, or spinal infantile paralysis—because of their recovery; while incurability is taken by the author as one of the prominent proofs of the essential "spinal infantile" paralysis. Who ever made his diagnoses from the results of treatment? and who ever, except our celebrated author, would at the same time deny the occurrence of rheumatic paralysis, and declare those favorable cases to be rheumatic? If further, spinal infantile paralysis depending on extravasation or exudation inside the vertebral canal is as incurable as he makes out, why is it that he still recommends the internal administration of absorbents? Have his one hundred and fifty cases proved the incurability so thoroughly, or have they informed him, as many of my own have me, of the possibility of removing exudations or extravasations from the vertebral canal with the same ease, or rather difficulty, as in other not very accessible localities?

Several other remarks and assertions of the author's are not more justified by facts. Thus it is not true, that the paralysis in the dental, or essential form, is more complete than in other kinds. The condition of the joint of the humerus, with its easy luxation and reposition, and the peculiar flabbiness of the muscles of the arm, are not at all characteristics of this disease. It is even observed in paralytic adults, with this exception, that both muscles and ligaments are more flaccid and stretch more easily in children than in adults. Nor is the absence of primary contraction in essential paralysis based on facts; the author himself does not believe in his own assertion as he mentions the paralytic form of scoliosis as depending on the asymmetrical contraction of the dorsal muscles. The real facts of the case are these, that the kind of paralysis of which I have spoken is seldom complete, but differs in the manner in which single muscles are affected. The equal power of antagonists is suspended, thus contraction is brought on by attempts at locomotion, and exercise in general as mentioned above. But this is not the only cause of contraction. For it is well known, especially in cerebral diseases, that the presence of a certain amount of extravasation, or effusion, gives rise to the symptoms of irritation, while a slight increase in the amount leads to depression and paralysis. We have no reason to believe this to be different in the spine. This early contraction depends on the presence of irritation, on the proportionate mildness or severity of the case, whether cerebral or spinal or peripheric, but not on the seat of the affection.

Nor is the seat of the affection so infallibly announced by the galvanic irritability of the muscles. For there are now a number of cases on record in which its absence in spinal diseases, and its intact condition in cerebral affections, were by no means constant and incontestable. Nor is the decrease of temperature greater in essential paralysis than in other forms; not the seat of the disease, but duration, the age at its first appearance, the atrophy, the nature and number of the affected (motory, sensitive, or vasomotory) nerves, and all the other causes of animal heat must be taken in regard, and may act just as thoroughly in cerebral as in spinal paralysis. And the atrophy itself, which has been said to show itself more rapidly in essential paralysis, depends not on the form or seat of the disease, but again on some of the abovementioned factors; on the rapidity of changes taking place in the infantile organism, in which infantile paralysis is mostly observed, and on the prevalence of fat at this age, which disappears more rapidly than any other tissue.

Finally, the assertion of Dr. von Heine, that the majority of cases of essential paralysis does not only occur in healthy and robust children, but that their very health and the amount of blood circulating in their organism predisposes them to extravasation and effusion in the vertebral canal, is, to say the least, somewhat unphysiological. The amount of blood contained in the system can be superabundant for a short time only; export and elimination increase in proportion to import and assimilation. If this is true in the adult, it is still more so in the child. On the other hand, it is a common observation that extravasation and effusion will mostly take place in anæmic individuals. It is true that scrofula, rhachitis, and other so-called dyscrasic processes, cannot be made responsible for the occurrence of essential paralysis, but still less both from theoretical reasons, and the results of my experience, I feel inclined to accuse health and a robust and vigorous constitution.—*Am. Med. Times.*

